# Anthropometric study of the knee in patients with osteoarthritis: intraoperative measurement versus magnetic resonance imaging

**DOI:** 10.1590/0100-3984.2016.0007

**Published:** 2017

**Authors:** Fabrício Bolpato Loures, Renato Janetti Carrara, Rogério Franco de Araújo Góes, Rodrigo Sattamini Pires e Albuquerque, João Maurício Barretto, André Kinder, Vinicius Schott Gameiro, Edson Marchiori

**Affiliations:** 1 MSc, Knee Surgeon at Hospital Santa Teresa, Petrópolis, RJ, Brazil.; 2 Member of the Brazilian Society of Orthopedics and Traumatology; MD, Intern in Knee Surgery at Hospital Santa Teresa, Petrópolis, RJ, Brazil.; 3 Member of the Brazilian Society of Knee Surgery; Head of the Professor Donato D'Ângelo Department of Orthopedics and Traumatology, Hospital Santa Teresa, Petrópolis, RJ, Brazil.; 4 PhD, Adjunct Professor at the Universidade Federal Fluminense (UFF), Niterói, RJ, Brazil.; 5 PhD, Member of the Center for Knee Surgery at the Instituto Nacional de Traumatología e Ortopedia, Rio de Janeiro, RJ, Brazil.; 6 MSc, MD, Radiologist at the Clínica Multimagem, Petrópolis, RJ, Brazil.; 7 PhD, Associate Professor at the Universidade Federal Fluminense (UFF), Niterói, RJ, Brazil; 8 PhD, Full Professor at the Universidade Federal do Rio de Janeiro (UFRJ), Rio de Janeiro, RJ, Brazil.

**Keywords:** Knee, Diagnostic imaging, Magnetic resonance imaging, Prostheses, Osteoarthritis, Anthropometry

## Abstract

**Objective:**

To compare intraoperative measurements of the knee with those obtained by magnetic resonance imaging, in order to validate the latter method for use in anthropometric studies.

**Materials and Methods:**

We studied 20 knees in 20 patients with osteoarthritis, all of whom underwent total arthroplasty between August and December of 2013. We took six measurements in the distal femur and two in the proximal tibia. Using the information system of the institution, we made the measurements on magnetic resonance imaging scans that had been obtained in the axial plane. Intraoperative measurements were obtained using a caliper, after the initial cuts made during the arthroplasty. The anatomical parameters determined by magnetic resonance imaging were the same as those determined by intraoperative measurement. The intraclass correlation coefficient was used in order to assess the level of agreement in anthropometric measurements of the knee performed by magnetic resonance imaging and by intraoperative measurement.

**Results:**

Statistical analysis revealed a highly significant correlation between the knee anthropometric parameters of the knee determined by intraoperative measurement and those determined by magnetic resonance imaging.

**Conclusion:**

The dimensions of osteoarthritic knees measured by magnetic resonance imaging were similar to those measured intraoperatively. Therefore, magnetic resonance imaging can be considered a reliable method for use in large-scale anthropometric studies that will allow the available implants to be adapted and improved.

## Abstract (pt)

**Objetivo:**

Comparar a mensuração do joelho realizada por meio de ressonância magnética com a medida obtida intraoperatoriamente, a fim de validar o método para estudos antropométricos.

**Materiais e Métodos:**

Foram estudados 20 joelhos em 20 pacientes portadores de osteoartrite submetidos a artroplastia total, entre agosto e dezembro de 2013. Realizaram-se seis medidas no fêmur distal e duas na tíbia proximal. As mensurações nas imagens foram feitas no plano axial, por meio do sistema informatizado da instituição. As mensurações intraoperatórias foram obtidas utilizando-se um paquímetro, após os cortes iniciais da artroplastia. Os parâmetros anatômicos foram os mesmos. O coeficiente de correlação intraclasses foi utilizado para avaliar a concordância nas medidas antropométricas de joelho realizadas pela ressonância magnética e pelo paquímetro de forma intraoperatória.

**Resultados:**

A análise estatística revelou que existe concordância altamente significativa entre as medidas antropométricas do joelho conseguidas por meio do uso do paquímetro intraoperatoriamente e pela ressonância magnética.

**Conclusão:**

A mensuração das dimensões dos joelhos osteoartríticos por meio de ressonância magnética apresentou-se como método semelhante à mensuração intraoperatória, sendo confiável para a realização de estudos antropométricos amplos que permitam a adequação e melhoria de implantes disponíveis.

## INTRODUCTION

Osteoarthritis is a degenerative disease that arises after long-term exposure of a genetically susceptible individual to an unfavorable environment^(1)^, causing deterioration of the entire joint^(2)^. It is the main cause of musculoskeletal disability in the elderly^(1)^. In Brazil, it currently affects approximately 12.4 million people^(3)^.

Because of certain anatomical and biomechanical factors, osteoarthritis primarily affects the knee^(1)^. The functional limitation caused by osteoarthritis and the progressive increase in life expectancy have generated an ever greater number of patients who are candidates for total knee arthroplasty. That procedure is capable of providing effective pain relief, restoring function and allowing a rapid return to daily activities^(4)^. However, it is a precision surgery, and differences between the size of the implant and the resected bone increase the complexity of the procedure^(5)^.

The main knee implants available on the market were based on anatomical studies performed in White individuals^(6)^, who present a mean height greater than that of the world population. In addition, the implants come in a limited number of sizes and require adaptations to their shape and kinematics^(7)^.

In order to improve the implants, knowledge of the anthropometry of the knees is fundamental, because the prosthesis is designed to mimic the natural articulation. Searching the literature in Portuguese and English, we identified only one study describing the anthropometric characteristics of the knee among individuals with arthrosis of the knee in Brazil, albeit with a limited number of individuals^(8)^.

The objective of the present study was to compare measurements of the knee obtained by magnetic resonance imaging (MRI) with those obtained intraoperatively, in order to validate the use of MRI in anthropometric studies.

## MATERIALS AND METHODS

We evaluated a convenience sample comprising 20 knees of 20 patients undergoing total knee arthroplasty between August and December 2013. The study was approved by the Research Ethics Committee of the Hospital Santa Teresa, in Petrópolis, RJ, Brazil, and all participating patients gave written informed consent. Patients with a history of knee fracture or surgery were excluded, as were those with bone loss that required grafting and those with varus or valgus deformity greater than 15°.

Patients underwent MRI of the affected knee at one week before admission. The examinations were performed in a 1.5 T scanner (Magnetom Essenza; Siemens, Erlangen, Germany), with the patient in the supine position and the knee relaxed in complete extension or with minimal flexion (< 15°) for maximum comfort. The following sequences were performed: sagittal proton density-weighted sequence with fat suppression-repetition time (TR) = 2800 ms; echo time (TE) = 35 ms; slice thickness = 4 mm; field of view (FOV) = 160 × 160 mm; matrix = 230 × 320-; sagittal T1-weighted sequence-TR = 540 ms; TE = 13 ms; slice thickness = 4 mm; FOV = 160 × 160 mm; matrix = 230 × 384]-; coronal proton density-weighted sequence with fat suppression-TR = 2040 ms; TE = 32 ms; thickness = 4 mm; FOV = 160 × 160 mm; matrix = 224 × 320-; and axial proton density-weighted sequence with fat suppression-TR = 3140 ms; TE = 35 ms; thickness = 4 mm; FOV = 160 × 160 mm; matrix = 192 × 320.

**Figure 1:**
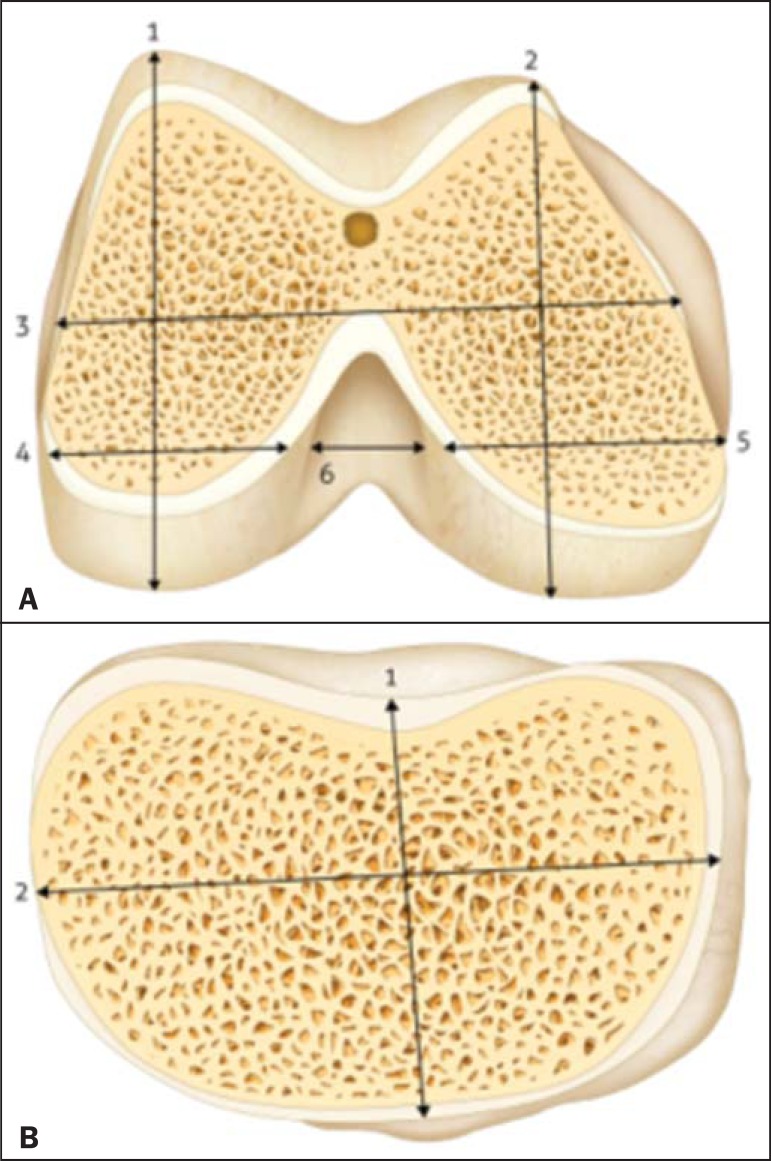
Schematic of the measurements. **A:** Distal right femur: 1, lateral condyle height; 2, medial condyle height; 3, total femur width; 4, lateral condyle width, 5, medial condyle width; 6, intercondylar width. **B:** Right proximal tibia: 1, anteroposterior width; 2, mediolateral width. Source: Loures et al.^(8)^.

Three metallic calipers were purchased and sent to the Brazilian National Institute of Metrology, Quality, and Technology, which confirmed the accuracy of the instruments. During the total knee arthroplasty, the surgeon used a caliper to take six measurements of the femur-lateral condyle height/width, medial condyle height/width, total mediolateral width, and total intercondylar width (Figure 1A)-and two measurements of the tibia-mediolateral width and anteroposterior width (Figure 1B). The dimensions were documented in millimeters (mm) after two measurements made by the principal investigator in order to rule out any interobserver variations. The arithmetic mean was used for analysis.

**Figure 2:**
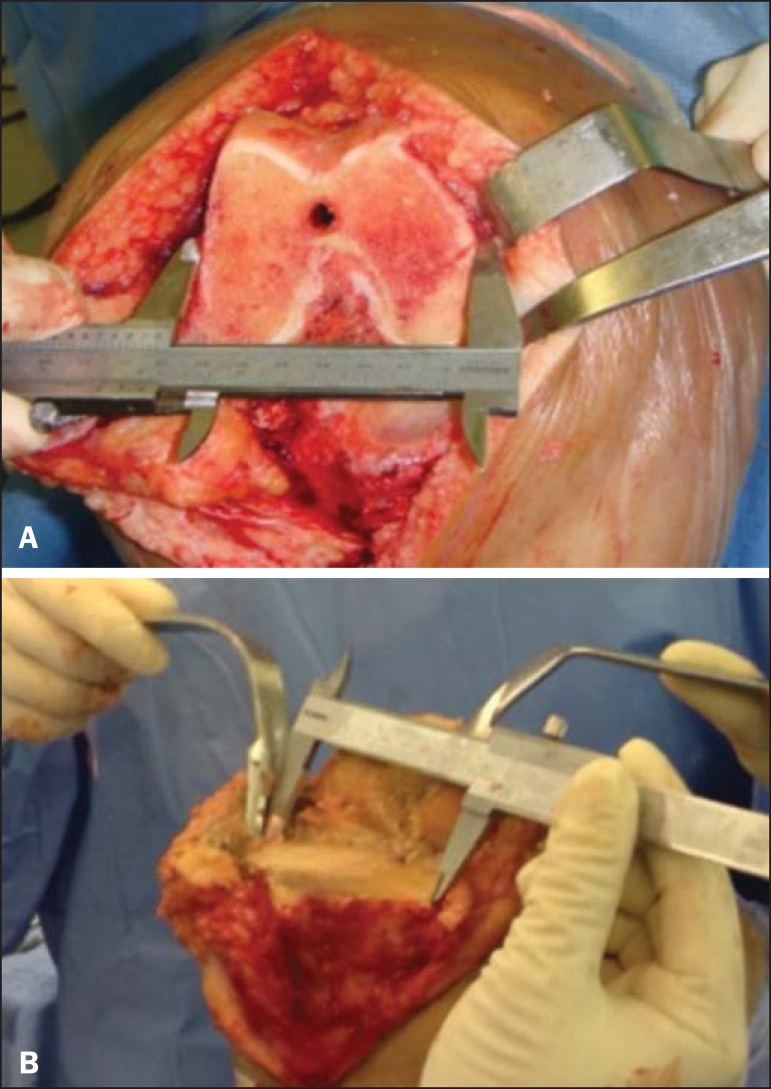
Intraoperative measurement. **A:** Right distal femur: measurement of the total width. **B:** Right proximal tibia: measurement of the mediolateral width.

The mediolateral distance width was measured at the level of the surgical transepicondylar axis. The widths of the femoral condyles were measured at 8 mm and 10 mm from the posterior articular surface, simulating the external rotation of the femur, for the lateral and medial condyles, respectively. The anteroposterior measurements were made considering the greatest distance between the posterior and anterior parts of the condyles. The anteroposterior width of the tibia was measured from the center of the insertion of the posterior cruciate ligament to the medial third of the patellar tendon. The mediolateral width was the greatest distance perpendicular to the anteroposterior width. Figure 2 shows the intraoperative measurement made with the caliper, which had previously been calibrated.

**Figure 3:**
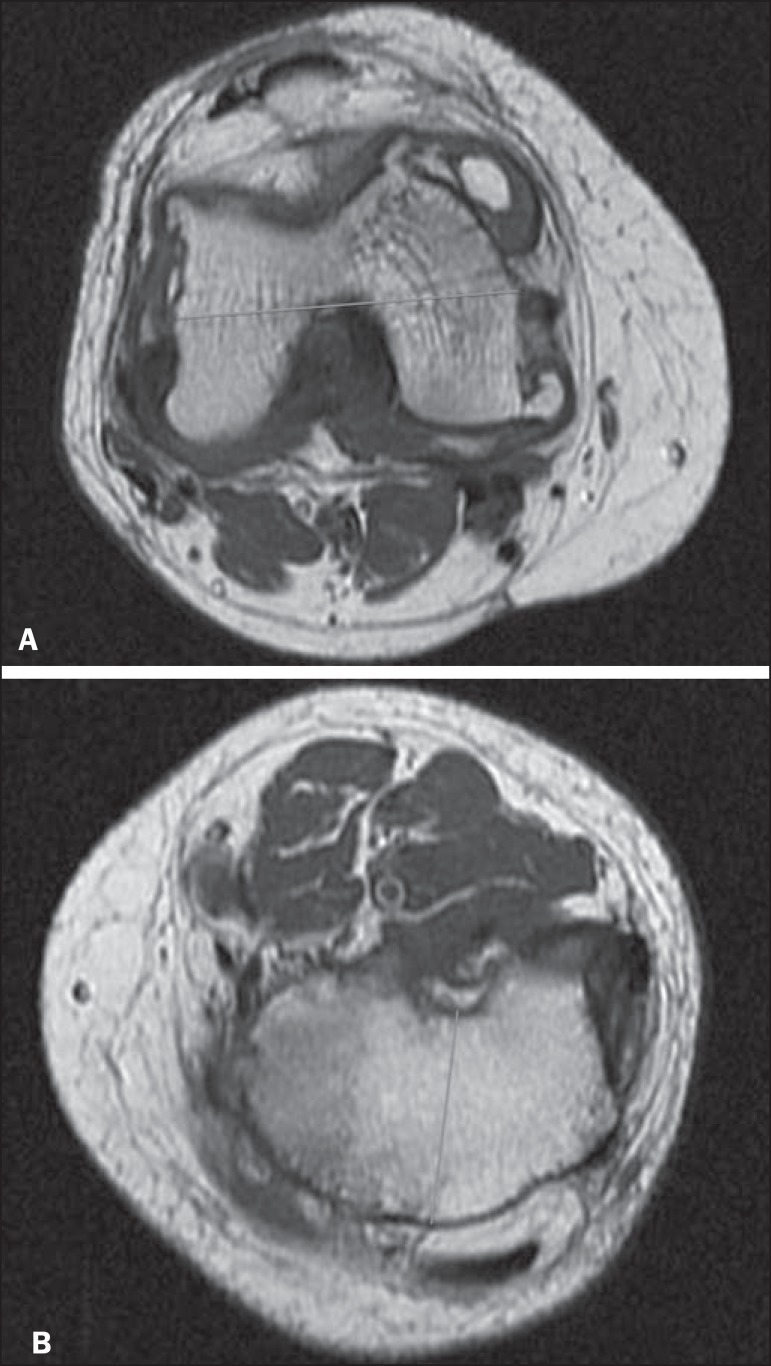
MRI measurement. **A:** Right distal femur: measurement of the total width. **B:** Left proximal tibia: measurement of the anteroposterior width.

While blinded to the identity of the patients, the surgeon made measurements on the MRI images using the same anatomical parameters. The measurements were made in the axial sequences, which allowed the simulation of the distal cuts of the femur and proximal cuts of the tibia. After two measurements, the arithmetic mean was calculated. Figure 3 shows the MRI measurements.

The measurements made by the two methods were digitized and sent to a biostatistician, who aggregated the data in order to analyze them.

The descriptive analysis is presented in the form of tables, the observed numerical data being expressed as means with standard deviations or as medians with minimums and maximums. The intraclass correlation coefficient (ICC) was used in order to evaluate the agreement between the anthropometric measurements of the knees made intraoperatively with the caliper and those obtained through MRI. The level of significance adopted was 5%. The statistical analysis was performed with the Statistical Package for the Social Sciences, version 18.0 (SPSS Inc., Chicago, IL, USA).

## RESULTS

The sample consisted of 20 patients with a clinical and radiographic diagnosis of arthrosis of the knee, all of whom were candidates for total knee arthroplasty.

**Figure 4:**
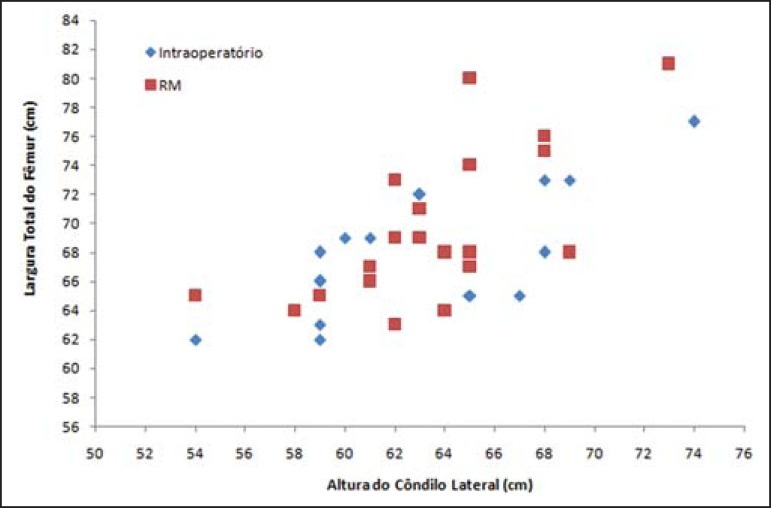
Comparison of measurements performed intraoperatively and by MRI.

The numerical values of the preoperative MRI measurements and intraoperative caliper measurements are shown in Table 1. The relationship between these two measurement modalities is demonstrated in a scatter plot (Figure 4).

**Table 1:** Measurements obtained intraoperatively (caliper method) and by MRI.

Parameter	Method	Mean	SD	Median	Min.	Max.
Total width of the femur	Caliper	67.9	4.3	68	62	77
	MRI	69.7	5.3	68	63	81
Lateral condyle width	Caliper	27.3	3.7	27	22	37
	MRI	26.9	3.0	26.5	23	34
Medial condyle width	Caliper	26.5	2.7	26.5	22	32
	MRI	26.6	2.4	26	23	33
Intercondylar width	Caliper	17.5	2.6	18	12	22
	MRI	16.9	2.7	16	12	23
Lateral condyle height	Caliper	63.4	4.8	63.5	54	74
	MRI	63.6	4.2	63.5	54	73
Medial condyle height	Caliper	62.9	4.6	62.5	54	73
	MRI	61.6	4.9	61	53	73
Mediolateral width of the tibia	Caliper	70.5	7.5	71	51	86
	MRI	71.3	8.2	70.5	52	89
Anteroposterior width of the tibia	Caliper	48.1	7.2	46	42	75
	MRI	48.5	6.9	47	42	74

SD, standard deviation; Min., minimum; Max., maximum.

The ICC values, the respective 95% confidence intervals, and the *p*-values resulting from the comparison between the two measurement methods are shown in Table 2.

**Table 2:** Comparison between the two measurement methods.

Measurement	ICC	95% CI	p-value
Total width of the femur	0.864*	0.693-0.943*	< 0.0001
Lateral condyle width	0.866*	0.697-0.944*	< 0.0001
Medial condyle width	0.677^†^	0.353-0.857^†^	0.0002
Intercondylar width	0.706^†^	0.400-0.871^†^	0.0001
Lateral condyle height	0.912*	0.794-0.964*	< 0.0001
Medial condyle height	0.840*	0.645-0.933*	< 0.0001
Mediolateral width of the tibia	0.958^‡^	0.898-0.983^‡^	< 0.0001
Anteroposterior width of the tibia	0.977^‡^	0.943-0.991^‡^	< 0.0001

ICC, intraclass coefficient correlation; 95% CI, 95% confidence interval.

* Excellent correlation;

† Good correlation;

‡ Near perfect correlation.

The statistical analysis revealed that, in our sample, the agreement between the anthropometric measures of the knee obtained through the preoperative use of MRI and those obtained through the intraoperative use of a caliper was highly significant. The level of agreement was excellent (ICC ≥ 0.80), except for the measurements of the medial condyle width and intercondylar width, both of which presented only good agreement (ICC = 0.68 and 0.71, respectively) (Table 2). Measurements of the mediolateral and anteroposterior width of the tibia showed an ICC close to 1 and a very narrow confidence interval, revealing a near-perfect correlation between the two methods (ICC = 0.958 and 0.977, respectively) (Table 2).

For the main anatomical parameters for arthroplasty (total width of the femur, lateral condyle height, mediolateral width of the tibia, and anteroposterior width of the tibia), even the weakest association between the two methods was excellent (ICC = 0.864). The power of the statistical test for a 5% level of significance, according to Cohen^(9)^, is above 90%.

## DISCUSSION

The knee is a joint with great driving force and one of the most complex joints the human body^(10)^. Osteoarthritis of the knee is a highly prevalent degenerative disease in developed countries^(1)^, and its incidence is increasing exponentially in developing countries, due to the growing number of obese individuals^(11)^, as well as to the aging of the population^(12)^. It is a chronic, progressive disease^(2)^ that evolves to deformity of the lower limbs, causing great functional limitation^(1)^ and leading many patients to seek surgical treatment.

Total knee arthroplasty requires accurate ligament balance and maximum coverage of the resected bone surfaces in order to achieve the best load distribution in the bone-implant relationship. Thus, the joint will remain stable throughout the range of motion and the implant will achieve its maximum lifespan^(13)^. Even with advances in surgical technique and prosthesis design, some studies have shown that a high proportion of patients submitted to total knee arthroplasty report dissatisfaction with the results^(14,15)^. Wylde et al.^(16)^ evaluated 250 knees in 242 patients who underwent total knee arthroplasty and found that 66% of the patients were “very satisfied” with the degree of pain relief and 52% returned to their normal day-to-day activities, although only 44% returned to their leisure activities. Implant incompatibility might be one of the factors that contribute to poor outcomes.

The function of the prosthesis is to mimic the original knee. Therefore, knowledge of the anthropometry of the population and its variables is fundamental for the production of appropriate implants. Although various authors have sought to describe the anthropometry of the knee of patients with arthrosis of the knee, the great majority of studies have focused on populations of White^(17-23)^ or Asian individuals^(6,23-27)^. We found a single study focusing on patients in Latin America^(8)^. That study involved intraoperative measurement but had a limited number of male patients.

Chaichankul et al.^(26)^ used MRI to study 200 knees of healthy Asian patients and found mismatches between implants and resected bones, as well as gender-related anatomical differences. Because their sample was composed of normal knees, it did not represent the anatomical alterations typical of arthrosis of the knee^(13)^. In addition, the authors did not calculate the reliability of the MRI measurements after simulation of the initial surgical cuts. In the present study, we sought to define which measurement methods correlate best with *in vivo* measurements in patients with osteoarthritis, in order to allow studies of noninvasive anthropometric measurements to be conducted on a larger scale.

Han et al.^(28)^ used MRI to study the dimensions of the knees of 535 volunteers. They divided those individuals into three groups, by age, and found significant differences among the young, middle-aged, and elderly. In addition to having received a clinical and radiographic diagnosis of osteoarthritis, all of the patients in our study were candidates for knee prosthesis implantation surgery and were over 60 years of age. We included elderly patients with knee pathologies, which was important because that group has its own anthropometric characteristics. In addition, the typical morphological alterations of osteoarthritis, such as decreased joint space, formation of osteophytes, and subchondral cysts, could be confounding factors in the image measurement, although that did not occur in the present study.

Shah et al.^(29)^ evaluated the tibial coverage of five models of prostheses for the knees of 150 patients in India. Measurements were performed after simulation of the proximal tibial cut in the axial MRI sequence, a method very similar to ours, with the exception of the rotational orientation, for which we used Akagi’s line^(30)^. We found that tibial measurements show near-perfect correlation, which allows this technique to be used without adjustments.

Vaidya et al.^(18)^ used computed tomography (CT) to study the dimensions of 86 knees in patients with arthrosis of the knee in India. They found great variability in the shape of the distal femur, and more than 40% of the patients presented dimensions smaller than those of the implants available. However, as described by Loures et al.^(31)^ and Voleti et al.^(32)^, CT does not consider the thickness of the articular cartilage, which limits the applicability of the method not only in the comparison with the implants but also in the definition of the anatomical standards.

Urabe et al.^(33)^ studied the distal femur in 100 women, of whom 70 were Japanese and 30 were White. Using measurements obtained by radiographic examinations, the authors found that the ethnicity and the height of the patient can both influence the shape of the distal femur. Radiography can present changes in magnification and technical imperfections^(32)^, both of which reduce its reliability for this type of study. Radiographic examination, like CT, uses radiation that can be harmful to the patient.

Cheng et al.^(13)^ suggested that the dimensions measured in the resected bones are more reliable for the comparison with the size and shape of the implants. We believe that intraoperative measurement, if performed by a surgeon with experience in arthroplasty and using a calibrated caliper, is the gold standard for anthropometry evaluation. However, it is an invasive method and one that is difficult to make widely available in a homogeneous manner. The MRI measurements, with simulation of the bone cuts, presented excellent correlations for the main parameters used in the choice of prosthesis size. Among the advantages of the method we emphasize the fact that it is a noninvasive examination that does not use radiation.

One limitation of our study is the lack of interobserver evaluation. However, the well-defined anatomical parameters allow the use of the technique by orthopedists and radiologists alike.

We conclude that the measurement of the dimensions of the osteoarthritic knees by MRI is a method comparable to that of intraoperative measurement, making it a reliable method for use in large-scale anthropometric studies, which could facilitate the adaptation and improvement of the available implants.
